# Ectopic Expression of a WRKY Homolog from *Glycine soja* Alters Flowering Time in *Arabidopsis*

**DOI:** 10.1371/journal.pone.0073295

**Published:** 2013-08-26

**Authors:** Xiao Luo, Xiaoli Sun, Baohui Liu, Dan Zhu, Xi Bai, Hua Cai, Wei Ji, Lei Cao, Jing Wu, Mingchao Wang, Xiaodong Ding, Yanming Zhu

**Affiliations:** 1 Plant Bioengineering Laboratory, Northeast Agricultural University, Harbin, Heilong Jiang, China; 2 Key Laboratory of Soybean Molecular Design Breeding, Northeast Institute of Geography and Agroecology, Chinese Academy of Sciences, Harbin, Heilong Jiang, China; 3 Department of Neurology, the University of Texas Southwestern Medical Center, Dallas, Texas, United States of America; Key Laboratory of Horticultural Plant Biology (MOE), China

## Abstract

Flowering is a critical event in the life cycle of plants; the WRKY-type transcription factors are reported to be involved in many developmental processes sunch as trichome development and epicuticular wax loading, but whether they are involved in flowering time regulation is still unknown. Within this study, we provide clear evidence that *GsWRKY20*, a member of WRKY gene family from wild soybean, is involved in controlling plant flowering time. Expression of *GsWRKY20* was abundant in the shoot tips and inflorescence meristems of wild soybean. Phenotypic analysis showed that *GsWRKY20* over-expression lines flowered earlier than the wild-type plants under all conditions: long-day and short-day photoperiods, vernalization, or exogenous GA_3_ application, indicating that *GsWRKY20* may mainly be involved in an autonomous flowering pathway. Further analyses by qRT-PCR and microarray suggests that *GsWRKY20* accelerating plant flowering might primarily be through the regulation of flowering-related genes (i.e., *FLC*, *FT*, *SOC1* and *CO*) and floral meristem identity genes (i.e., *AP1*, *SEP3*, *AP3*, *PI* and *AG*). Our results provide the evidence demonstrating the effectiveness of manipulating *GsWRKY20* for altering plant flowering time.

## Introduction

In higher plants, a phase transition from vegetative to reproductive development is one of the most important events in their life history [1,2]. This transition is tightly coordinated through a diverse array of signaling networks that integrate various endogenous and exogenous signals [3]. Flowering time is a key trait in adaptation, as it is vital for reproductive success. *Arabidopsis thaliana* contains at least four flowering pathways that are responsive to these cues: the photoperiod pathway monitors changes in day length; the gibberellin pathway plays a promotive role in flowering under non-inductive photoperiods; the vernalization pathway senses the prolonged exposure to low temperature; and the autonomous pathway mediates flowering by perceiving plant developmental status [3–5]. Most recently, an endogenous pathway that adds plant age to the control of flowering time has been described [6]. Several genes, such as *CONSTANS* (CO), *FLOWERING LOCUS T* (*FT*), SUPPRESSOR OF OVEREXPRESSION OF CO 1 (SOC1), and FLOWERING LOCUS C (FLC) have been identified as key components in these flowering signal pathways [3]. *CO*, which encodes a zinc-finger transcriptional activator, controls the timing of flowering by positively regulating two floral integrators, *FT* [7] and *SOC1* [8]; *FLC*, a flowering repressor gene, also acts as an upstream regulator gene of *FT* and *SOC1* [9]. Moreover, these flowering integrators have been shown to exhibit both overlapping and independent functions in the determination of flowering time and they integrate signals from multiple flowering pathways and their expression levels eventually determine the exact flowering time [3,10].

During the signaling of flowering regulation, a number of transcription factors (TFs) are included. MADS-domain TF family is one of the most important TF families that function in flowering regulation. Among the floral transition genes, *FLC*, *SOC1*, *APETALA1* (*AP1*), *APETALA3* (*AP3*), *PISTILLAT* (PI), AGAMOUS (AG) and *SEPALLATA3* (*SEP3*) are members of the MADS-box gene family [11]. Furthermore, members of other transcription factor families have been identified for their role in the regulation of floral MADS-domain proteins and /or other flowering time genes directly or indirectly [11], such as NACs [12], MYBs [13], DREBs [14].

WRKY proteins are a class of DNA-binding transcriptional factors which contain one or two highly conserved WRKY domains typically having a conserved WRKYGQK motif at N-termini as well as a C2H2 or C2HC zinc-finger structure which is distinct from other known zinc-finger motifs at C-termini [15]. To date, numerous WRKY proteins have been experimentally identified from more than 10 plant species, and it has become clear that WRKY TFs play key roles in responses to biotic and abiotic stress along with various hormones [16–18]. Some WRKY genes also have been reported to be involved in developmental processes. For example, *SUSIBA2* [19] and *MINISEED3* [20] playing roles in the regulation of seed development; also, *Testa Glabra 2* (*TTG2*)/*AtWRKY44* playing a role in trichome development and mucilage and tannin synthesis in the seed coat [21], and *OsWRKY89* increasing epicuticular wax loading [22]. We have recently reported that the *GsWRKY20*, isolated from wild soybean, played a role in the developmental processes of stomata and cuticle, mediated ABA signaling and improves the drought tolerance [23].

In this paper, we will report a novel physiological function of *GsWRKY20* in planta, the ectopic overexpression of *GsWRKY20* in *Arabidopsis* (Col-0) accelerating flowering time. qRT-PCR analysis showed that overexpression of *GsWRKY20* altered the transcriptional profiles of the genes which were involved in flowering control, implicating that *GsWRKY20* may play an important role, not only in in stress [23] but also in flowering transition. Furthermore, we conclude that *GsWRKY20* accelerates *Arabidopsis* flowering may mainly through an autonomous pathway.

## Materials and Methods

### Sequence analysis of GsWRKY20

Sequence alignments were performed with ClustalW. Phylogenetic analysis was performed using MEGA 4.1. The amino acid sequence of GsWRKY20 and other homologues were retrieved and compared to decipher their relationship. The accession numbers of the genes were listed in Information S1.

### Plant Materials and Growth Conditions

The landrace *G07256* of wild soybean (*Glycine soja*) was obtained from Jilin Academy of Agricultural Sciences (Changchun, China). The seeds were sown in soil in 10-liter pots in a growth chamber and grown at a consistent air temperature of 25°C and 16 h light/8 h dark cycles. The light source SON-T ARGO 400 W generated constant illumination of 30000 lx. At the seedling stage, each pot was thinned to 6 plants. These plants were grown till the cotyledons opened or until the unifoliates fully expanded. The plants were then treated with different photoperiods of SD (8 h light/16 h dark) or LD (16 h light/8 h dark). All other parameters for plant growth and treatments were described by Zhu et al [24].

*Arabidopsis thaliana* (ecotype Col-0 background) seeds were obtained from the Nottingham Arabidopsis Stock Centre (NASC). The *GsWRKY20* over-expression lines have been described previously [23]. *Arabidopsis thaliana* seeds were pretreated at 4°C for 3 days and sown in pot soil or on half-strength Murashige and Skoog (MS)-agar plates (0.6 g L-1 MES pH 5.8 and 0.8% w/v agar, hereafter referred to as 0.5× MS-agar plates) for germination and growth at 22°C air temperature, 100 µmol photons m^-2^ s^-1^and 60% relative humidity.

### Gene Expression Analyses

*GsWRKY20* tissue-specific expression levels in *G. soja* cv G07256 plants were analyzed by quantitative real-time RT-PCR (qRT-PCR). Total RNA was isolated from root, trifoliate leaf, stem, flower bud, and pod. To analyze the diurnal expression of *GsWRKY20* in wild soybean leaves, the fully developed young trifoliate leaves from the plants grown under SD or LD were sampled every 4 h starting at dawn for a total of 20 hours. For analyzing the expression of flowering regulating genes, 10-day-old to three-week-old wild type (WT) and *GsWRKY20* overexpression line 28 transgenic *Arabidopsis* seedlings were harvested from 0.5× MS agar plates at the given indicated time intervals for qRT-PCR and microarray (the *Arabidopsis* ATH1 Genome Arrays, Affymetrix) assays. All microarray experiments including data analysis were carried out as described previously [25]. For the expression analysis of the *FLC*, *CO*, *SOC1* and *FT* at different growing days, *GsWRKY20*ox line 28 and WT plants were grown in soil, and from the first occurrence of bolting to the last flowering, the leaves were sampled every day. All of the above samples were taken for three biological replicates at the indicated time after treatments.

### Quantitative real-time RT-PCR

Total RNA was extracted using RNeasy Plant Mini Kit (Qiagen, Valencia, CA, USA) and on-column DNA digestion was performed to remove any contamination of genomic DNA using RQ1 RNase-free DNase (Promega, USA). RNA quality was verified by agarose gel electrophoresis, and cDNAs were synthesized by using oligo d(T)_18_ reverse primer from 2μg of total RNA in a total volume of 20 μL by using the SuperScript™ III Reverse Transcriptase kit (Invitrogen, Carlsbad, CA, USA).

Prior to the qRT-PCR assays, the quality of the cDNA samples were assessed by PCR using *GAPDH* specific primers for wild soybean, and *ACTIN2* specific primers for *Arabidopsis* . qRT-PCR reactions were carried out in 96-well (25 μL) format by using the SYBR Green Master Mix (Invitrogen, Carlsbad, CA, USA), and were performed in an Agilent Technologies Stratagene Mx3005p Real-Time PCR system. *GAPDH* and *ACTIN2* were used to normalize all values in the qRT-PCR assays in wild soybean and in *Arabidopsis* , respectively. All of the reactions were performed in biological triplicates using RNA samples extracted from three independent plant materials and the gene-specific primers designed using Primer5 software were listed in Information S1. Expression levels for all candidate genes were determined using the 2^-△△CT^ method, relative transcript levels were calculated and normalized as described previously [26]. The locus of the candidate genes were listed in Information S1.

### Flowering time

After 3-day cold stratification, the WT and *GsWRKY20*ox seeds were sown and germinated in pot soil. Plants were grown under different conditions till flowering. Flowering time was measured by counting the number of rosette leaves and the number of days to flower (when the floral buds are visible) [27]. For the LD experiment, the plants were grown under 16/8 h light/dark photoperiod. For the SD experiment, the plants were grown under 16/8 h dark/light photoperiod. For gibberellic acid (GA_3_, Sigma-Aldrich) and paclobutrazol (PAC) treatments, when the two cotyledons fully opened, the plants were sprayed with 100 μM GA_3_ twice a week until flowering, or watered with 37mg/L PAC solution once a week [28].

For vernalization treatments, the seeds were transferred to 4°C for one month before they were continued to grow at 22°C under the light illumination of 50 μmol photons m^-2^ s^-1^. The time spans seed germination (measured as the time until stem elongation [bolting] was observed) and was tabulated starting from the beginning of the first day at the higher temperature (22°C) [29,30].

## Results

### Sequence analysis of GsWRKY20

**Figure 1:**
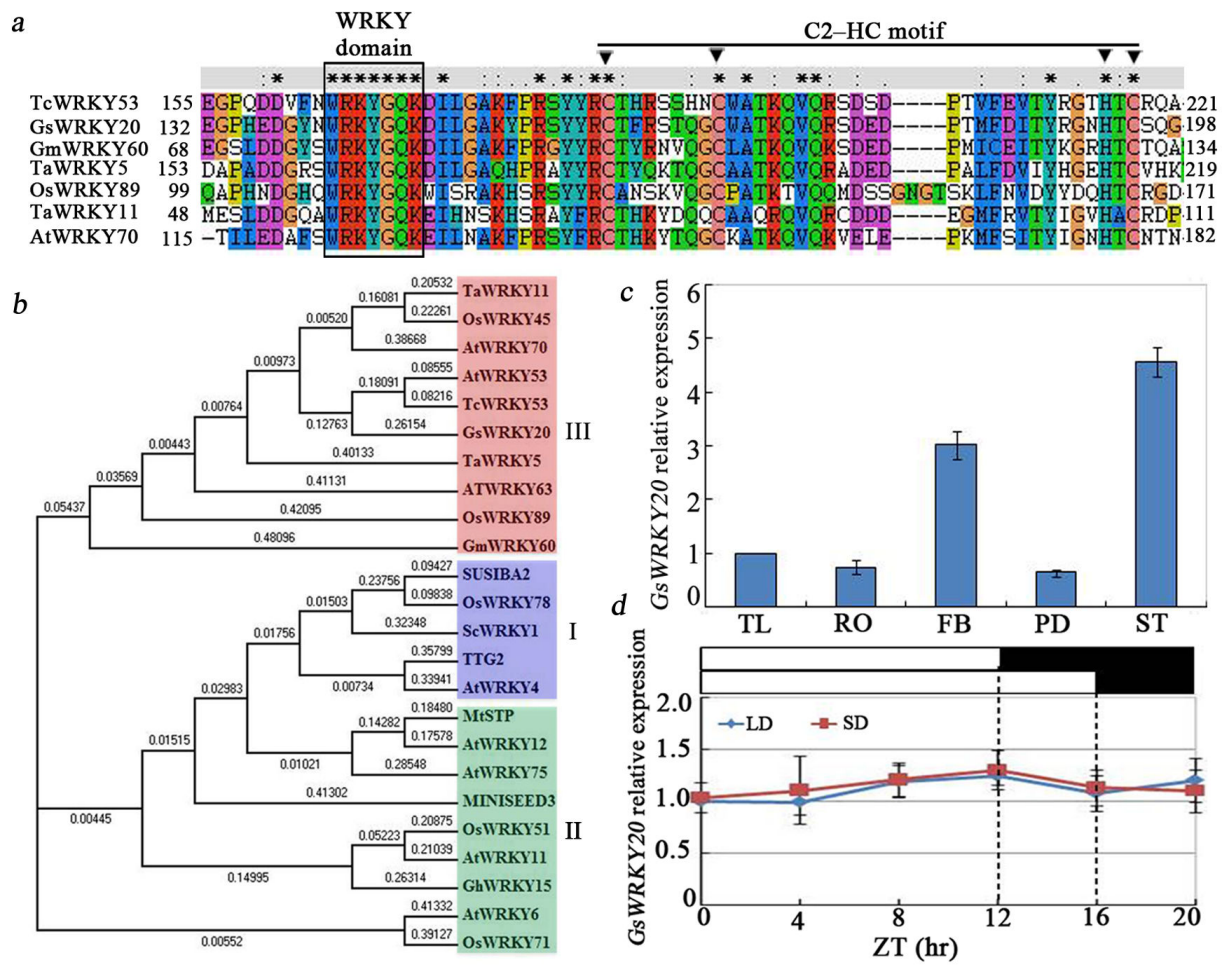
Sequence and expression analysis of *GsWRKY20*. (**a**) Amino acid sequence alignment of WRKY domains among GsWRKY20 and other type Ⅲ WRKY TFs, Sequences were aligned using ClustalW, and gaps were introduced to maximize alignment, filled triangle marks the cystine and histidine in the C2HC-type zinc finger domain. (**b**) The phylogenetic tree of the WRKY TFs. The phylogenetic tree was constructed using MEGA 4.1. Total 24 WRKY proteins from *Oryza*, *Arabidopsis, Gossypium, Medicago, Triticum aestivum, Glycine max, Thlaspi caerulescens* and *Solanum* were selected to construct the phylogenetic tree. (**c**) Tissue-specific expression analysis of *GsWRKY20* by real-time quantitative PCR (qRT-PCR). Tissues included trifoliate leaf (TL), root (RO), stem tip (ST), flower bud (FB), and pod (PD). Expression of *GAPDH* was used as an internal control. The experiment included three fully independent biological repeats, and three technical repeats and the mean value is shown. (**d**) qRT-PCR analysis of *GsWRKY20* diurnal expression under SD and LD. Trifoliate leaves were sampled every 4 h at 21 DAE. White and black bars at the top represent light and dark phases, respectively. Relative transcript levels were analyzed by qRT-PCR and normalized by *GAPDH*. The experiment included three fully independent biological repeats, and three technical repeats and the mean value is shown.

In our previous study, *GsWRKY20* was identified as an ABA signaling regulator and drought stress response gene [23]. Based on the sequence analysis, the predicted GsWRKY20 protein contains one conserved WRKY domain and a C2HC-type zinc finger motif (C-X_7_-C-X_23–27_-H-X_1_-C) (Figure 1a). These conserved motifs suggest that GsWRKY20 belongs to the type Ⅲ WRKY subgroup. An alignment analysis by ClustalX revealed that GsWRKY20, together with TcWRKY53, AtWRKY70, OsWRKY89, GmWRKY60, TaWRKY5 and TaWRKY11, belongs to the type Ⅲ WRKY TFs. GsWRKY20 shares 81.8%, 70.9%, 53.8%,76.4%, 74.5, 54.5 identity in WRKY domains and 36.2%, 23.1%, 18.5%, 28.8%, 24.3% and 17% identity in complete sequences with the above six WRKY TFs (Figure 1a), indicating that apart from the WRKY domains the sequences of WRKY proteins are highly divergent.

In order to gain insights into the evolutionary pathway of GsWRKY20 among the other WRKY TF orthologs which are involved in development processes, a total of 24 WRKY proteins from *Oryza*, *Arabidopsis* , *Gossypium* , *Medicago* , *Triticum aestivum*, *Glycine max*, *Thlaspi* *caerulescens* and *Solanum* were retrieved, and a phylogenetic tree was constructed using Neighbor–Joining method (Figure 1b). The result revealed that these WRKY proteins were classified into three groups. GsWRKY20 was more closely related to TcWRKY53, AtWRKY53, AtWRKY70 and OsWRKY45 in group Ⅲ. Among these proteins, OsWRKY45 [31] and TcWRKY53 [32] are involved in abiotic stress, while AtWRKY53 [33] and AtWRKY70 [34] are involved in development process. The phylogenetic analysis indicates that GsWRKY20 probably has a distinct role in plant development and should be investigated further.

### Expression patterns of GsWRKY20

The *Glycine soja* cv 07256 seedlings were planted and maintained under LD conditions until the unifoliates were fully expanded. Hereafter, one set of plants was kept growing under LD conditions and another set was kept growing under SD conditions. *GsWRKY20* expression profiles were detected by qRT-PCR using *GAPDH* as a reference. The data showed that *GsWRKY20* expression was observed in almost all tissues, including the root, leaf, flower, pod and inflorescence stem (Figure 1c). Notably, the *GsWRKY20* expressed significantly higher in flowers and inflorescence stems than in roots and leaves, suggesting that *GsWRKY20* may function in reproductive development. To know if *GsWRKY20* gene expression has diurnal circadian rhythm, the trifoliate leaves (15 DAE) were sampled every 4 h. Expression of *GsWRKY20* under SD and LD conditions did not exhibit diurnal circadian rhythm (Figure 1c), suggesting that *GsWRKY20* was not regulated by circadian clock genes. Therefore, *GsWRKY20* may have a role in reproductive development, but it is not involved in photoperiodic pathway.

### Over-expression of GsWRKY20 in Arabidopsis promotes early flowering

**Figure 2:**
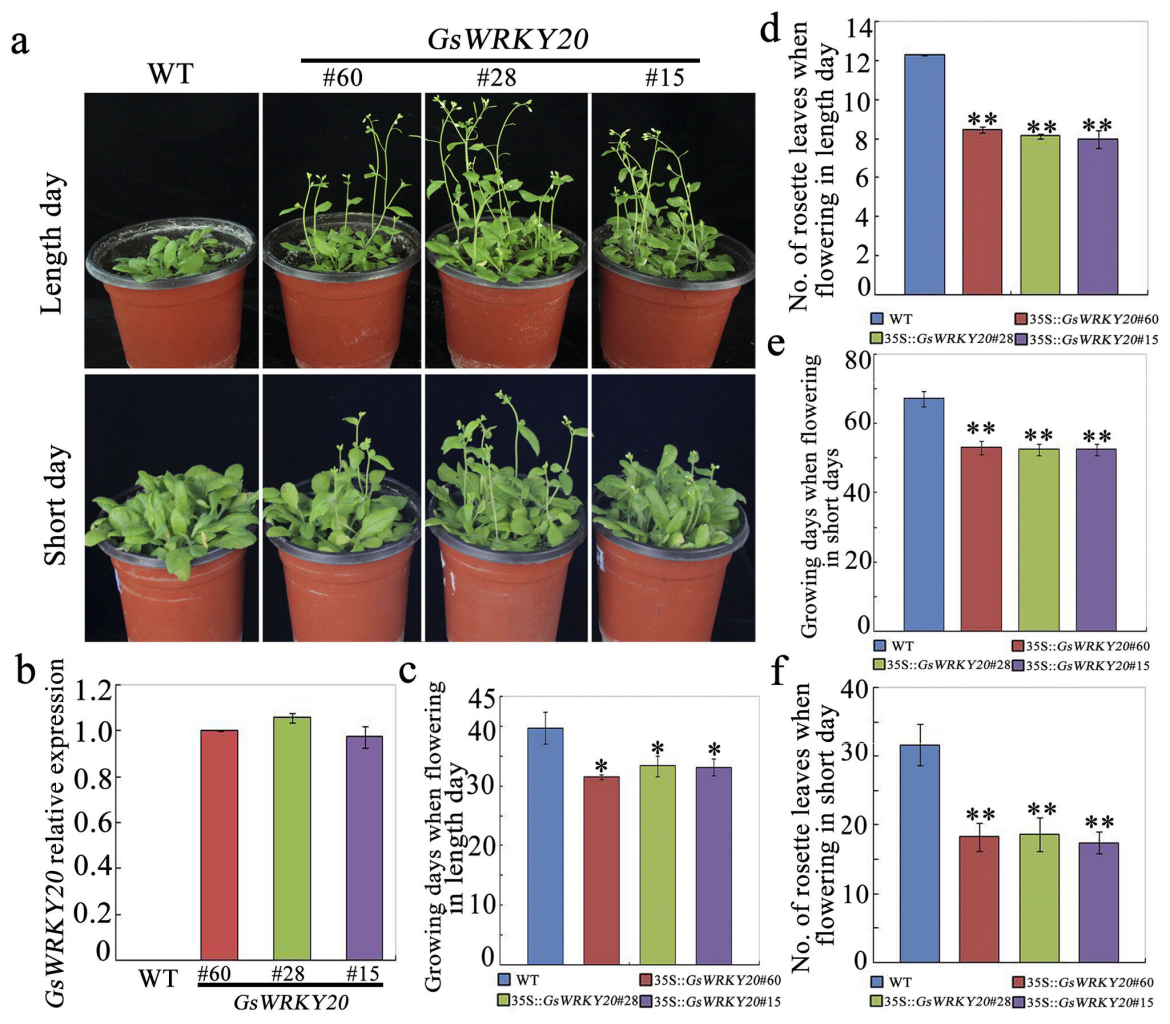
Over-expression of *GsWRKY20* in Arabidopsis accelerates plant flowering. (**a**) Flowering time of *GsWRKY20*ox plants was accelerated under both LD and SD conditions. (b) qRT-PCR analysis of *GsWRKY20* transcript levels in WT and the three homozygous 35S :: *GsWRKY20* lines. Expression of *ACTIN2* was used as an internal control. The experiment included three fully independent biological repeats, and three technical repeats and the mean value is shown. (**c**) Average flowering time of WT and *GsWRKY20*ox plants at the time of flowering under LD conditions. (**d**) Average rosette leaf numbers of WT and *GsWRKY20*ox plants at the time of flowering under LD conditions. (**e**) Average flowering time of WT and *GsWRKY20*ox plants at the time of flowering under SD conditions. (**f**) Average rosette leaf numbers of WT and *GsWRKY20*ox plants at the time of flowering under SD conditions. All values in (c, d, e, f) are means (±S.E.) from three independent experiments (At least 30 seedlings per experiment). Data were analyzed statistically using the t-test, Asterisk and double asterisks indicate significant differences from the corresponding WT at 0.01 <*P*< 0.05 and *P*< 0.01, respectively.

We have recently reported that the *GsWRKY20* is intimately related with ABA-mediated drought tolerance [23]. Notably, we also found that all of the three independent homozygous T_3_ transgenic lines (line 60, line 28, and line 15) with high expression of *GsWRKY20* (Figure 2b) also showed early flowering phenotype compared to WT plants grown under the 16/8 h (light/dark) photoperiod (LD) (Figure 2a). The *GsWRKY20* over-expression lines, which were referred as *GsWRKY20*ox plants, flowered with an average of 32.1-day vegetative growth and 8.2 leaves at flowering time under LD, whereas WT plants flowered with an average of 39.7-day vegetative growth and 12.3 leaves at flowering time (Figure 2c, d). The early flowering promotive role of *GsWRKY20* suggested that it is an important component plant reproductive development

Plant flowering time is regulated by many genes mainly via four genetically distinguishable pathways, i.e., autonomous, photoperiod, vernalization, and GA pathways [3]. In order to further identify in which pathways *GsWRKY20* may be involved, WT and *GsWRKY20*ox plants were given different treatments (photoperiods, vernalization, and GA_3_).

Under the SD condition, the average rosette leaf numbers of WT and *GsWRKY20*ox plants at the time of flowering were 32.6 and 18.7 respectively, and *GsWRKY20*ox plants flowering occurred almost more than two weeks ahead of the WT plants (Figure 2e, f). These results demonstrate that *GsWRKY20* overexpression can promote precocious flowering independent of the photoperiod.

**Figure 3:**
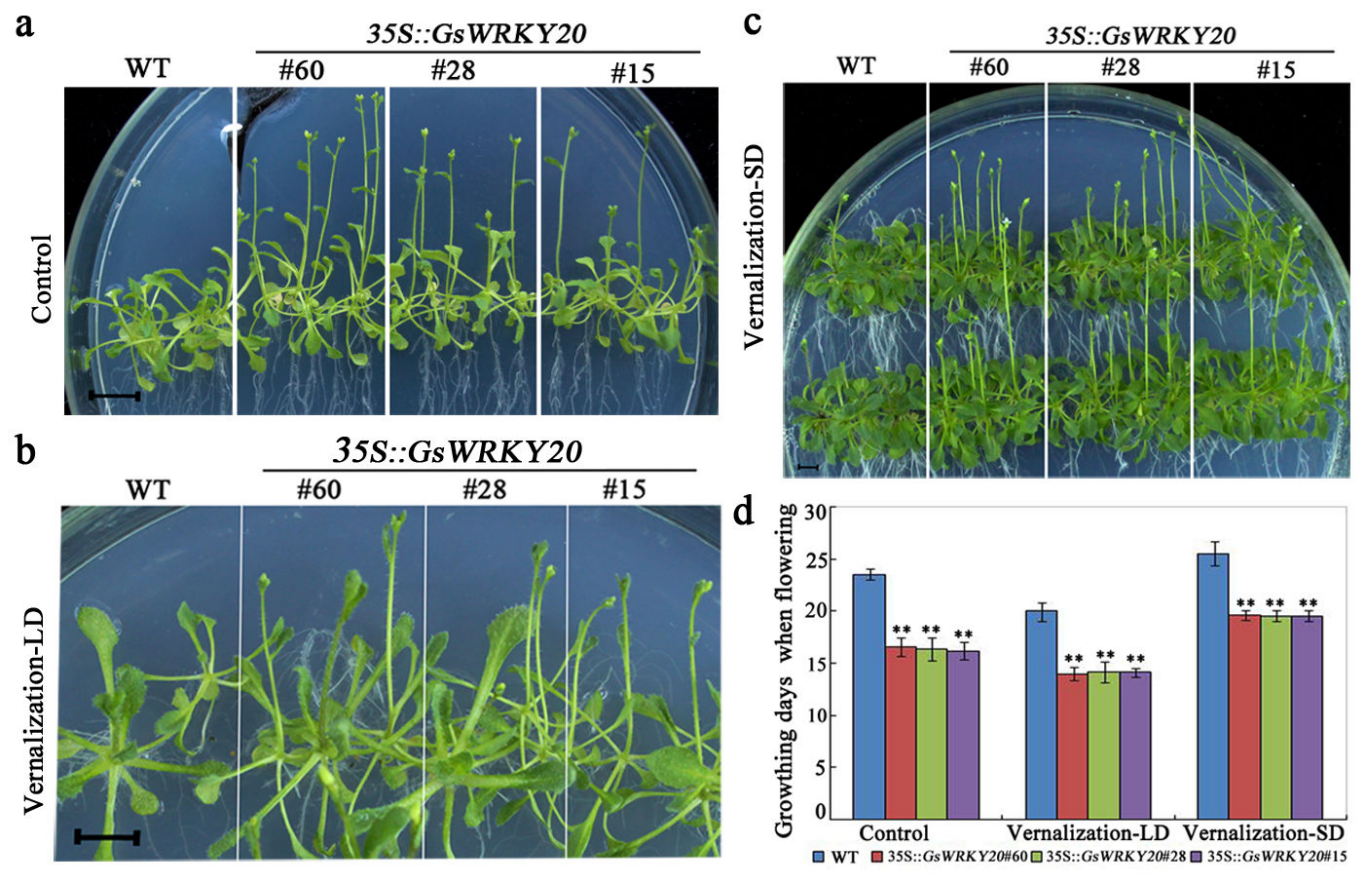
*GsWRKY20*ox plants exhibited early flowering under vernalization treatment. (**a**) Flowering phenotype of *GsWRKY20*ox plants which were germinated on 0.5 × MS agar plates was accelerated under LD conditions, scale bar: 0.5cm. (**b**) Flowering phenotype of *GsWRKY20*ox plants after one month of vernalization under LD. The seedlings were vernalized for one month at 4°C before transferred to LD photoperiods and grown at 22°C. (**c**) Flowering phenotype of *GsWRKY20*ox plants after one month of vernalization under SD. The seedlings were vernalized for one month at 4°C before transferred to SD photoperiods and grown at 22°C. (**d**) Average flowering time of WT and *GsWRKY20*ox plants which were described in (a, b, c) ; Times to flowering were determined as the time until stem elongation [bolting] was observed. All values are means (±S.E.) from three independent experiments (at least 18 seedlings per experiment). Data were analyzed statistically using the t-test, double asterisks indicate significant differences from the corresponding WT at *P* < 0.01.

The functional activity of *GsWRKY20* in the vernalization pathway was also investigated under the LD and SD conditions. Our results showed that *GsWRKY20*ox plants and the WT plants responded normally to vernalization, vernalization treatment promoted flowering of *GsWRKY20* over-expression lines and the WT plants compared to the normal condition, but *GsWRKY20*ox plants exhibited earlier flowering after vernalization treatment (Figure 3b, c). After one month of vernalization, *GsWRKY20*ox plants which were germinated and grown on 0.5 × MS agar plates flowered more than about 6 days ahead of the WT plants both in LD and SD conditions (Figure 3d).

**Figure 4:**
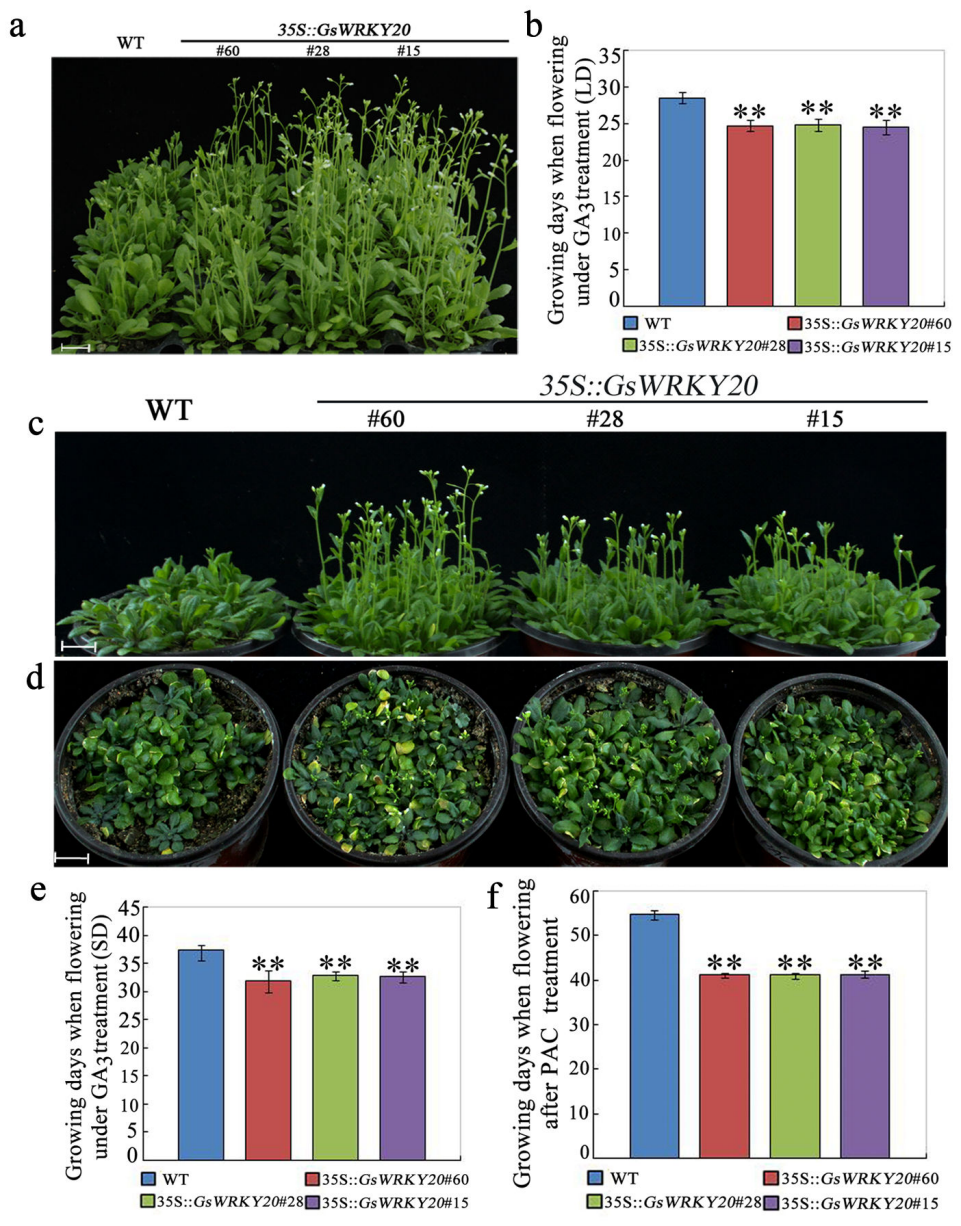
*GsWRKY20*ox plants exhibited early flowering under GA_3_ treatment. (**a**, **c**) Flowering phenotype of WT and *GsWRKY20*ox plants which were treated with GA_3_ under LD (a) and SD (c) condition. The plants were sprayed with 100 μM GA_3_ twice a week when the two cotyledons fully opened, scale bar: 1.0 cm. (**d**) Flowering phenotype of WT and *GsWRKY20*ox plants under PAC treatment. The plants were watered with 37mg/L PAC concentrated solution once a week under LD condition, scale bar: 1.0 cm. (**b**, **e**, **f**) Average flowering time of WT and *GsWRKY20*ox plants which were described in (a, c, d) at the time of flowering; Times to flowering were determined as the time until stem elongation [bolting] was observed. All values are means (±S.E.) from three independent experiments (at least 30 seedlings per experiment). Data were analyzed statistically using the t-test, double asterisks indicate significant differences from the corresponding WT at *P* < 0.01.

In order to determine the involvement of *GsWRKY20* in the GA-regulated flowering pathway, *GsWRKY20*ox and WT plants, were sprayed with 100 μM GA_3_ twice a week. *GsWRKY20*ox and WT plants treated with GA_3_ flowered significantly earlier than their non-GA_3_-treated controls under both LD (Figure 4a) and SD conditions (Figure 4c). After exogenous GA_3_ application, *GsWRKY20*ox plants flowered more than 4 days ahead of the WT plants in LD conditions (Figure 4b) and 7 days ahead of the WT plants in SD conditions (Figure 4e). In LD conditions, the WT and *GsWRKY20*ox plants began flowering with an average of 12.3 and 8.2 total rosette leaves, respectively, and the mean total rosette numbers decreased to 10.26 and 6.51 after GA_3_ application (shown in Information S1). Under SD, the mean rosette leaves numbers also decreased from 32.6 and 18.7 to 18.15 and 13.16 (shown in Information S1), respectively. The experimental results show that *GsWRKY20*ox plants exhibited earlier flowering after spraying with exogenous GA_3_. To further ascertain this conclusion, we watered the *GsWRKY20*ox and WT plants with the GA biosynthesis inhibitor paclobutrazol (PAC) to block endogenous GA biosynthesis under LD condition. We found that the *GsWRKY20*ox plants still exhibited earlier flowering than the WT plants (Figure 4d). However, after the PAC treatment, the vegetative growth phases of *GsWRKY20*ox and WT plants were prolonged to 41.7 and 54.3 days, respectively (Figure 4f). These results suggest that *GsWRKY20* should not be involved in the GA-induced flowering pathway.

In all, these results indicated that *GsWRKY20* was involved in the regulation of flowering time through regulatory pathways other than the above three pathways.

### GsWRKY20 promotes the expression of flowering-related genes

**Figure 5:**
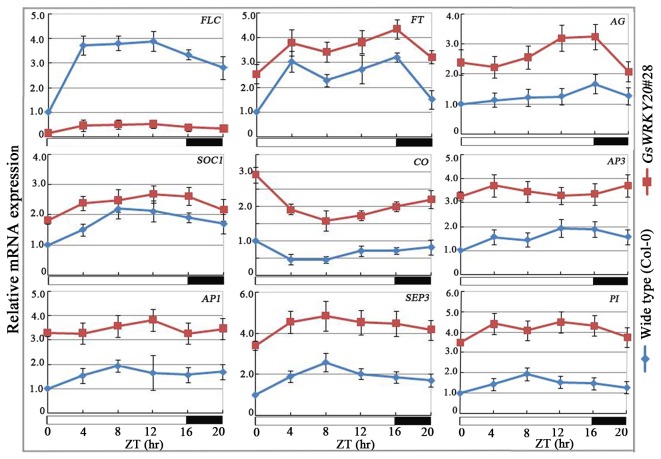
The effect of *GsWRKY20* over-expression on the transcription of *FLC*, *FT*, *SOC1*, *CO*, *AP1*, *SEP3, AG, PI* and *AP3*. Ten-day-old WT and *GsWRKY20* ox seedlings were harvested every 4 h during LD condition, and mRNA expression level was determined by qRT-PCR. Each value is the mean ±SE of three independent measurements, error bars represent the standard deviation (n=3). White and black bars at the bottom represent light and dark phases, respectively. ZT, Zeitgeber.

**Figure 6:**
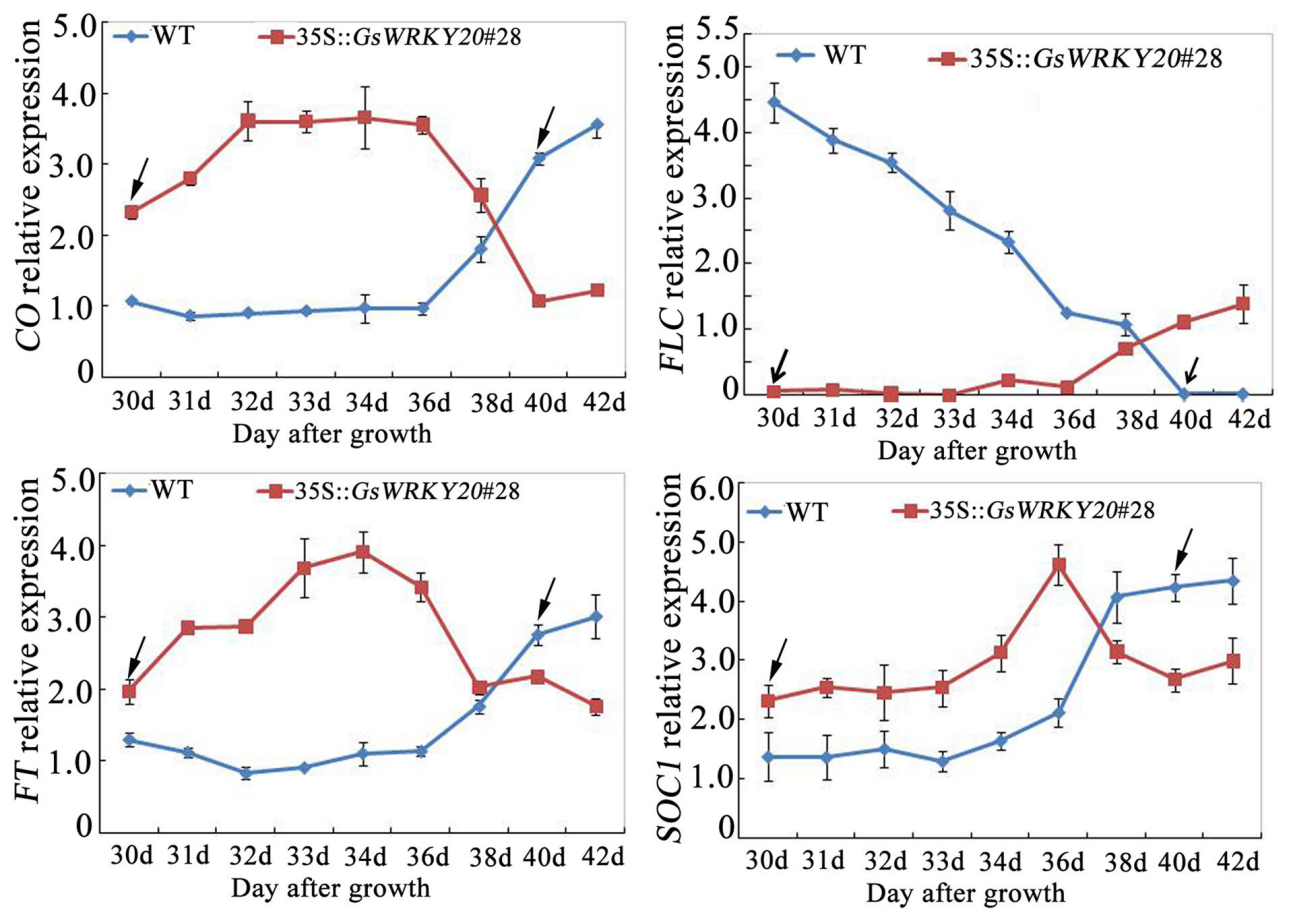
Expression analysis of the *CO*, *FLC, FT* and *SOC1* in *GsWRKY20*ox and WT plants during the flowering transition stage. The experiment included three fully independent biological repeats. Arrows indicate the number of growth days from seed germination to flowering (bolting 2 cm), respectively.

The results mentioned above strongly suggested that *GsWRKY20* may be involved in the autonomous flowering pathway. Considering the genetically distinguishable pathways that regulate the flowering time of *A. thaliana* are integrated by the expression of flowering pathway integrators, to explore the evidence to support the hypothesis that *GsWRKY20* may be involved in the autonomous flowering pathway, the expression levels of the genes which are involved in the determination of flowering time were monitored by qRT-PCR. *FLC* is a key regulator gene of the autonomous pathway [3], so we first analyzed the expression of *FLC*, and found that *GsWRKY20* significantly suppressed *FLC* expression (Figure 5), and on the other hand we found that *GsWRKY20* promoted the expression of another major flowering-related gene, *CO* (Figure 5). Since *FLC* negatively but *CO* positively regulate *FT* and *SOC1* [9], we measured the expression levels of *FT* and *SOC1* and the results showed that both of them indeed exhibited higher levels in *GsWRKY20*ox plants than the WT plants (Figure 5), and the increased expression of *FT* and *SOC1* was independent of sampling time during the diurnal cycle, suggesting that both of them may be implicated in *GsWRKY20* signaling. The flower identity gene *SEPALLATA3* (*SEP3*) is known to interact with *AP1*, and its over-expression can hasten flowering [35]. In *GsWRKY20*ox plants, we found the expression levels of *SEP3* and *AP1* were also increased (Figure 5). AP3 and PI are closely related MADS domain proteins that are thought to act as obligate heterodimers [36]. SEP3, AP1 and AG were identified as interaction partners of AP3 and PI [11], so *AP3*, *PI* and *AG* were further determined and we found the expression levels of them were also elevated in *GsWRKY20*ox plants (Figure 5). Some studies have shown that the expression levels of *FLC*, *CO*, *SOC1* and *FT* in Arabidopsis exhibit different during the flowering transition stage [2,3], so we further analyzed the expression of these four flowering integrator genes in the WT plants and *GsWRKY20*ox plants under different growing days by qRT-PCR, and the results showed that the expression levels of *FLC*, *CO*, *SOC1* and *FT* in WT and *GsWRKY20*ox plants indeed exhibited different during the flowering transition stage, the flowering repressor *FLC* was down-regulated and the flowering activators *CO, SOC1* and *FT* were all up-regulated much more earlier in *GsWRKY20* over-expression lines than in WT (Figure 6).

**Figure 7:**
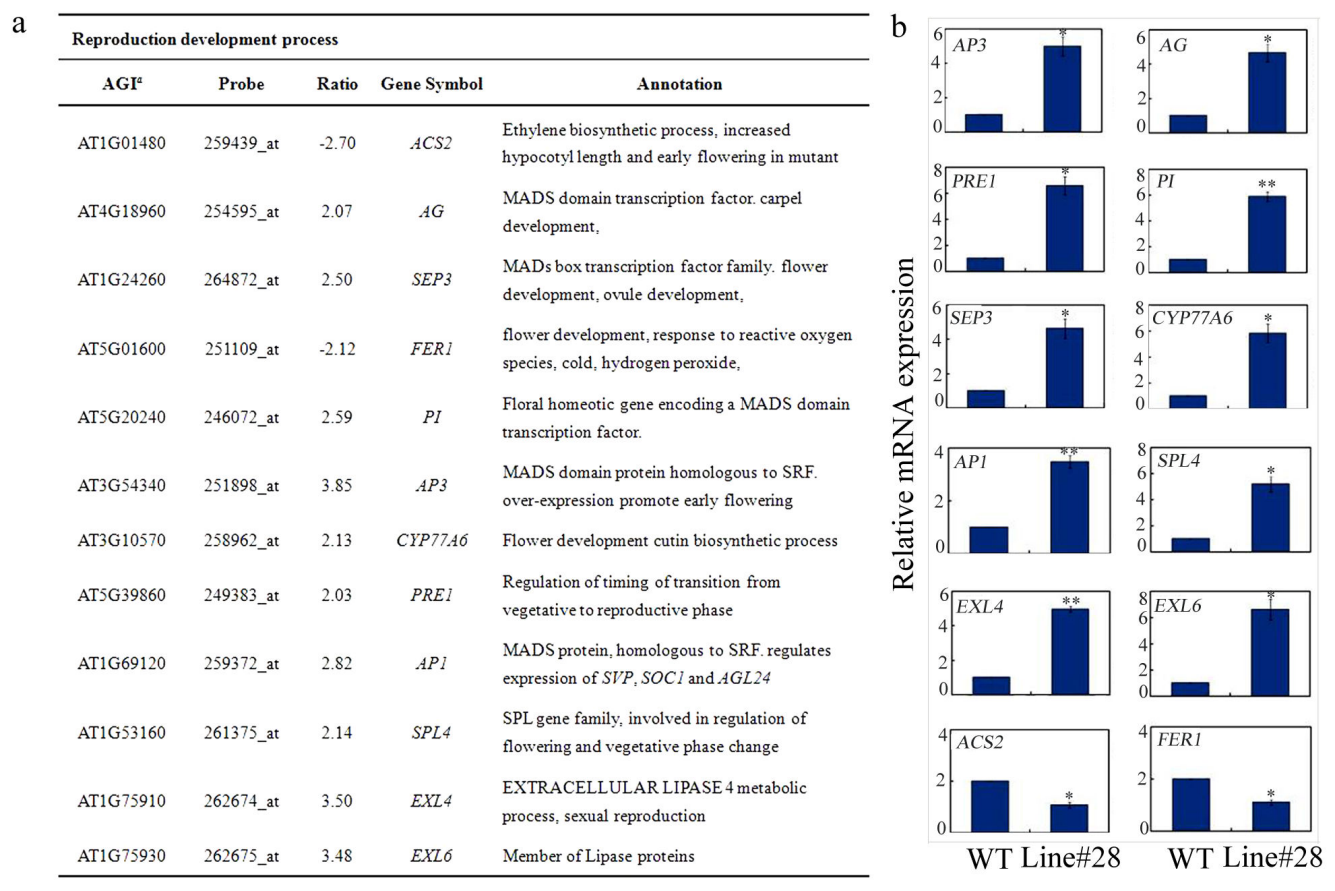
The differentially expressed flowering-related genes in *GsWRKY20*ox plants revealed by microarray. (**a**) The differentially expressed flowering-related genes in *GsWRKY20*ox plants. Genes which were up or down-regulated (>2-fold) in the *GsWRKY20*ox plants compared with the WT are listed. The *P* values were corrected for multiple testing using FDR methodology. The group of genes was classified based on their biochemical functions. AGI, Arabidopsis Genome Initiative number; FC, fold changes. (**b**) Expression validation of the differentially expressed flowering-related genes. Total RNAs were extracted from three-week-old whole plants grown on 0.5 × MS-agar. Transcript levels relative to *ACTIN2* are presented for each treatment. Each value is the mean ±SE of three independent measurements. Significant differences from WT are denoted by one, or two stars corresponding to *P* < 0.05, and *P* < 0.01, respectively by Student’s t test.

To explore more data about the role of *GsWRKY20* in plant flowering, we performed microarray assays using the Affymetrix ATH1 Gene Chip. Differentially expressed genes were identified after statistical analysis, approximately 301 genes were up or down-regulated (≥ 2-fold change) in the *GsWRKY20*ox lines (shown in Information S2). A major functional category of the differentially expressed genes showed that some of them were involved in ABA signalling, stress regulation, and we also found that there were 12 flowering-related genes were up or down-regulated (≥ 2-fold change) in the *GsWRKY20*ox plants. The expression levels of these 12 genes were further confirmed by qRT-PCR. *ACS2* and *FERI* negative regulators of flowering time, were down-regulated, whereas *AP3*, *AG*, *AP1*, *SPL4*, *PRE1*, *PI*, *EXL4*, *EXL6*, *SEP3* and *CYP77A6* positive regulators of flowering development, were up-regulated in *GsWRKY20* over-expression plants (Figure 7), suggesting the transcription factor GsWRKY20 may act as an upstream regulator to orchestrate the expression of the above flowering-related genes to control plant flowering pattern.

## Discussion

WRKY TFs have been reported to regulate plant various developments, but no data is available about whether WRKY TFs are involved in flowering time regulation. In the present study, our data provide clear genetic evidence for the function of *GsWRKY20* in controlling floral initiation. We found that *GsWRKY20* acts as a positive regulator of flowering, since the transgenic plants over-expressing *GsWRKY20* demonstrated early flowering compared to WT. Flowering time is known to be coordinated by at least four pathways, namely autonomous, photoperiod, vernalization, and GA pathways [3]. *GsWRKY20*ox plants flowered earlier than the WT under both LD and SD conditions. Flower development of *Arabidopsis* is promoted by LD condition and delayed by SD condition [3]. In our case, *GsWRKY20*ox plants grown under SD condition flowered significantly earlier than WT plants; however, flower production of both WT and transgenic plants was delayed compared to the plants grown under LD condition. Thus, over-expression of *GsWRKY20* can accelerate flower formation, but cannot overcome the photoperiodic effect, suggesting that *GsWRKY20*ox plants were still sensitive to photoperiod and *GsWRKY20* should be independent of photoperiod flowering pathway. Vernalization flowering pathway indicates that low temperature treatment of germinating seed can induce early flowering [30], to judge whether *GsWRKY20* was involved in vernalization flowering pathway mainly based on whether vernalization will suppress the early flowering phenotype of the *GsWRKY20*ox plants, although the transgenic acceptor Col-0 is capable to flower without vernalization, we demonstrated that *GsWRKY20*ox plants and the WT plants responded normally to vernalization, vernalization treatment promoted flowering of *GsWRKY20*ox plants and the WT plants compared to the normal condition, but *GsWRKY20*ox plants still flowered much more earlier than WT plants both in LD and SD conditions, so vernalization cannot suppress the early flowering phenotype of *GsWRKY20* transgenic lines. Since the *GsWRKY20*ox plants showed a normal response to vernalization and exhibited earlier flowering, a role of *GsWRKY20* in vernalization flowering pathway was excluded. With this approach, many flowering-related Arabidopsis lines of Columbia (Col-0) background were examined whether they were involved in vernalization flowering pathway. For example, with this approach, the Col-0 background mutants sr45-1 [37] and syp22-1 [38] were demonstrated that they were not involved in vernalization flowering pathway. On the other hand, all *GsWRKY20*ox plants flowered earlier than WT plant after sprayed with exogenous GA_3_ or watered with the GA biosynthesis inhibitor PAC, suggesting that *GsWRKY20* is excluded from GA_3_ pathway. From these data, we can conclude that *GsWRKY20* was involved in the regulation of flowering time through regulatory pathways other than the above three pathways.

Most recently, an endogenous pathway that adds plant age to the control of flowering time has been described [6], and it is independent on the *FT* expression and does not affect the expression levels of *FLC*, so, the likely involvement of *GsWRKY20* in the autonomous pathway was further verified by the down-regulation of *FLC* in *GsWRKY20*ox plants in this study (Figure 7a), Mockler et al (2004) reported that the *FLC* transcript level was up-regulated in mutants deficient in the autonomous pathway of *Arabidopsis* [39]. We also found that the expression of *CO* was significantly higher in *GsWRKY20*ox plants, and the previous study has shown that over-expression of *CO* gene promoted early flowering under any day length [3]. CO is a transcriptional activator and FLC is a suppressor of *FT* and *SOC1*. Opposite to the role of *FLC*, we found the expression of *FT* and *SOC1* were up-regulated in *GsWRKY20*ox plants.

The family of MADS domain transcription factors play important roles in floral transition, specification of floral organ identity and floral determinacy [40,41]. Flowers develop from floral meristems (FM) that arise in the peripheral zone of the reproductive SAM [41], which is also called the inflorescence meristem (IM). The four different floral organs that are developed from the FM are specified by combinations of different types of MADS transcription factors. Five major floral homeotic MADS-domain proteins (AP1, AP3, PI, AG and SEP3) have been proved playing the positive roles in floral initiation and development. These MADS-domain proteins interact with each other, such as SEP3, AP1, and AG were identified as interaction partners of AP3 and PI [42]. SEP3 was also known to interact with AP1 and AG, and its over-expression hastens flowering, genetic analysis revealed that *AP1* and *SEP3* could regulate the expression of *AP3*, *PI* and *AG* [43]. As a kind of florigen, the elevated FT protein moves from the leaf to the apex, where it promotes the expression of *AP1* and *SEP3* [3]. Hence, the different integrators directly or indirectly activate floral meristem identity genes *AP1* and *SEP3*, and then launch the expression of a series of genes and mark the beginning of floral organ formation [3]. In our experiments, the expression levels of *AP1*, *AP3*, *PI*, *AG* and *SEP3* revealed by qRT-PCR and microarray were obvious up-regulated in the *GsWRKY20*ox plants, indicating that *GsWRKY20* may be a critical regulator of these five genes or the function of *GsWRKY20* may require the participation of these five proteins.

The early flowering phenotype (the average number of growing days and rosette leaves at flowering time) of the three homozygous T_3_ transgenic lines were essentially the same, so, to be sure, ectopic expression of *GsWRKY20* attributed to the earlier flowering phenotype of these over-expression plants. And the *GsWRKY20* levels in these three transgenic lines did not show any significant difference each other (Figure 2b), so only one transgenic line was used to detect the expression levels of the flowering regulation genes which might be regulated by *GsWRKY20*. Our previous study also suggest that although expression levels of the downstream genes which regulated by the overexpressed gene in different transgenic lines may be not exactly the same, but they usually did not exhibit significant differences each other [44,45], and a lot of researchers also just used only one transgenic line to detect the expression levels of the downstream genes which might be regulated by the overexpressed gene [46–49].

As the over-expression of *GsWRKY20* resulted in altered expression patterns of flowering, we postulated that GsWRKY20 functioned as a transcriptional regulator, acting as a master regulator of downstream flowering-related genes. Our current findings appear to rule out a role in transcriptional repression, insofar as a fusion of *GsWRKY20* and the GAL4 DNA binding domain could induce *LacZ* expression in yeast [23], implicating that GsWRKY20 may act as an activator of gene transcription, which is consistent with most current reported WRKY TFs [50,51]. *GsWRKY20*-over-expressing *Arabidopsis* characterizes this protein as a putative negative regulator of *FLC*. In this regard, GsWRKY20 could be indirectly involved in the regulation of *FLC* possibly via transcriptional activation of a number of negative regulators. However, transcriptional activation assay using yeast system only provides an indirect evidence that GsWRKY20 possesses transcription activation function, so we cannot figure out the possibility that GsWRKY20 has the other regulation mechanisms in plant.

WRKY TFs can regulate many types of genes directly through binding to W-box (TTGACC/T) [51] or non-W box sequences [19,52–55], and they are also found to regulate various genes indirectly. For examples, AtWRKY63 could bind to the W-box so as to directly regulate the expression of *ABF2*, and indirectly control the expression of *RD29A* and *COR47* [50]. There are also some reports of WRKY proteins binding to non-W box sequences. OsWRKY13 can bind to the PRE4 element (TGCGCTT) as well as to W-box [54], and barley HvWRKY46 [53] can bind to both W boxes and a sugar-responsive (SURE) element (TAAAGATTACTAATAGGAA) whereas tobacco (*Nicotiana tabacum*) NtWRKY12 appears to bind a SURE-like element but not the W box [19]. On the other hand, although MINI3 (WRKY10) can bind to W-boxes in the *MINI3* and *IKU2* promoters, but MINI3 cannot activate the transcription of *MINI3* and *IKU2* [56]. So whether WRKY proteins can bind to W-box is not the necessary requirement for their ability to regulate the expression of downstream genes.

**Figure 8:**
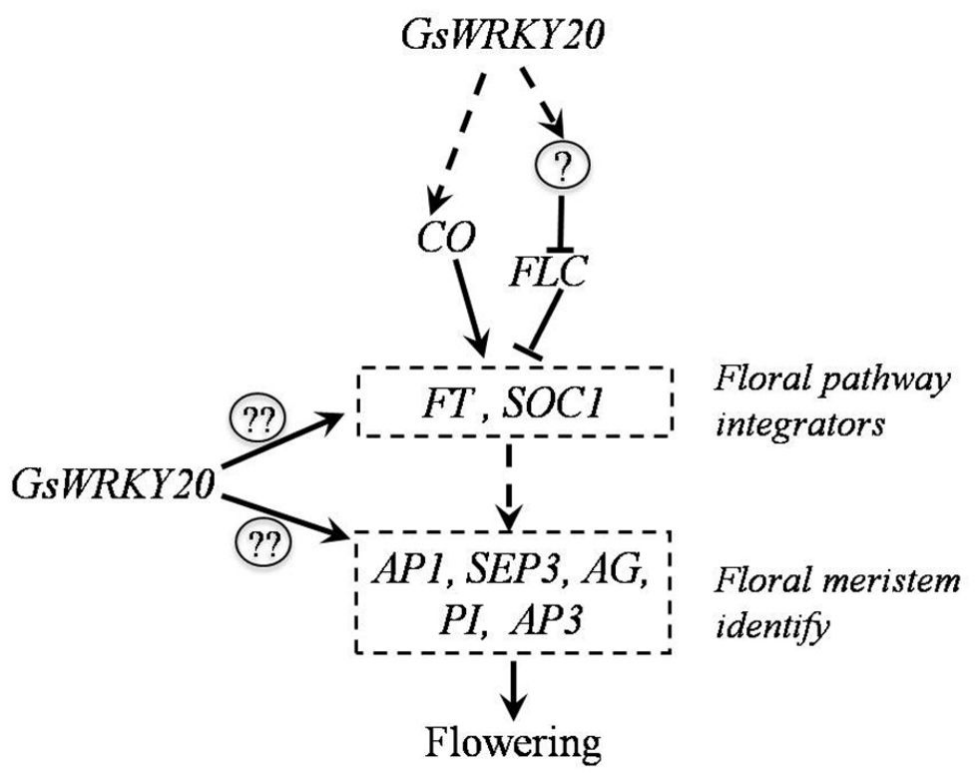
Proposed model for the role of *GsWRKY20* in the regulation of *Arabidopsis* flowering time. The symbol <?> indicates an unknown factor or signaling cascade that may repress the *FLC* gene expression, and the symbol <??> indicates the possibility that *GsWRKY20* directly regulate the expression of the floral pathway integrators *FT* and *SOC1* and the floral meristem identity genes *AP1*, *AP3*, *PI*, *AG* and *SEP3*. The straight dotted lines represent directly or indirectly regulation; the solid straight lines represents directly regulation. Arrows denote the positive effects; and lines terminated with a bar denote inhibitory effect.

Thus, we established a model to reveal a missing link in the *GsWRKY20-*mediated flowering signaling pathway between the primary signaling events to downstream gene expression (Figure 8). In this model, GsWRKY20 protein acts as a positive e regulator of floral development in *Arabidopsis* . GsWRKY20 promotes flowering may mainly via the autonomous pathway by indirectly inhibiting *FLC* which is a suppressor of flowering-promoting factors, FT and SOC1; and on the other hand, *GsWRKY20* enhances the *CO* expression directly or indirectly, which subsequently promotes the expression of *FT* and *SOC1*. The different integrators will then directly or indirectly strengthen the expression of floral meristem identity genes *AP1*, *SEP3*, *AP3*, *PI* and *AG* mark the beginning of floral organ formation. In this model, however, some other unknown factors or signaling cascades involved in the repression of *FLC* expression may exist and are directly or indirectly regulated by *GsWRKY20*.

Finally, it is noteworthy that, in this model, the underlying mechanisms of several important steps remain open questions. Whether *GsWRKY20* directly enhance the expression of *CO*? And whether *GsWRKY20* directly regulates the expression of the floral pathway integrators *FT* and *SOC1* and the floral meristem identity genes *AP1*, *SEP3*, *AP3*, *PI* and *AG*? Further research to answer these questions will shed new light on *GsWRKY20*-mediated flowering signal transduction.

## Supporting Information

Information S1 **Included are three sections, that is, Gene-speciﬁc primers used for RT-PCR assays, Locus or accession number of the genes, and Average rosette leaf numbers of WT and *GsWRKY20*ox plants at the time of flowering after GA_3_ treatment.**
(PDF)

Information S2 **Up or down-regulated genes in the *GsWRKY20*ox line28 (≥2 Fold Change, compared with the WT).**
(XLS)
