# Supplementary material for: Ectopic Expression of a WRKY Homolog from Glycine soja Alters Flowering Time in Arabidopsis
Source: PLoS One. 2013 Aug 26;8(8):e73295. doi: 10.1371/journal.pone.0073295 (PMC3753250; doi:10.1371/journal.pone.0073295)
Supplement: Information S1 — Included are three sections, that is, Gene-speciﬁc primers used for RT-PCR assays, Locus or accession number of the genes, and Average rosette leaf numbers of WT and GsWRKY20ox plants at the time of flowering after GA3 treatment. (PDF) [file pone.0073295.s001.pdf]

Locus or Accession number of the genes

Gene symbol	Gene locus or Accession number	Gene symbol	Gene locus or Accession number
GAPDH	DQ355800	*AtWRKY12*	AT2G44745
ACTIN2	AT3G18780	*AtWRKY53*	AT4G23810
FLC	AT5G10140	*AtWRKY63*	AT1G66600
CO	AT5G15840	*AtWRKY70*	AT3G56400
SOC1	AT2G45660	*AtWRKY75*	AT5G13080
FT	AT1G65480	*TaWRKY5*	EU665434.1
AP1	AT1G69120	*TaWRKY11*	EU665440
SEP3	AT1G24260	*OsWRKY45*	GQ331932
AP3	AT3G54340	*OsWRKY61*	BK005064
AG	AT4G18960	OsWRKY71	AB190817.1
PI	AT5G20240	*OsWRKY78*	BK005212
SPL4	AT1G53160	*OsWRKY89*	AY781112.1
PRE1	AT5G39860	*GmWRKY60*	EU375357
EXL4	AT1G75910	*GhWRKY15*	HQ651070.1
EXL6	AT1G75930	*TcWRKY53*	EF053036
CYP77A6	AT3G10570	*ScWRKY1*	AY366389
ACS2	AT1G01480	*SUSIBA2*	AY323206.1
AtWRKY4	AT1G80840	*MINISEED3*	NM_104436.2
AtWRKY6	AT1G62300	*MtSTP*	HM622067
AtWRKY11	AT4G31550	*TTG2*	AF516172

Gene-specific primers used for RT-PCR assays

Gene name	Primer Sequence (5' to 3')
GsWRKY20	Forward:GGCCATCAAGGATCACCATAAT-3'<br>Reverse: GGCTCCAAGGATGTCTTTCTGA-3'
GAPDH	Forward: GACTGGTATGGCATTCCGTGT<br>Reverse: GCCCTCTGATTCCTCCTTGA
ACTIN2	Forward:TTACCCGATGGGCAAGTC<br>Reverse: GCTCATACGGTCAGCGATAC
FLC	Forward: GCTTTCTGTTCTCTGTGACGCA<br>Reverse: CTCCAGTTGAACAAGAGCATCG
CO	Forward: GCTCCCACACCATCAAACTTACT<br>Reverse: CGATTGGCAGAGTGAACTTGAG
SOC1	Forward: ACACAAATAGATGAAACGAGGAAAG<br>Reverse: GATAAAAACCTAACCAGGAGGAAGC
FT	Forward: ACAATCAACACAGAGAAACCACCT<br>Reverse: CCTGAGGTCTTCTCCACCAATC
AP1	Forward: GCAAGCAATGAGCCCTAAAGAG<br>Reverse: AGTGCGGATGTGCTTAAGAGC
SEP3	Forward: GCATGCTTCGGACACTGGA<br>Reverse: GGCCTCTCTTGAAGGCACATT
AP3	Forward: GAGTGTTTGGACGAGCTTGACA<br>Reverse CGCGAACGAGTTTGAAAGTGTT
AG	Forward: CAACCGTTTGATTCACGGAA<br>Reverse: GGCGGATGAGTAATGGTGATTG
PI	Forward: AAAATCTGATGGCTGTCGAGC<br>Reverse: CCATCTGGTGGTCTCGGACT
SPL4	Forward: GTAGCATCAATCGTGGTGGC<br>Reverse: CTTCGCTCATTGTGTCCAGC
PRE1	Forward: CTTGGTCGTTTCCTTTCAGCA<br>Reverse: TTGATTATCGGAGATCCTTGGAG
EXL4	Forward: ACAGCAGGTCCTTCAAGAGCA<br>Reverse: CCCAACGGTATCACTCCCATTA
EXL6	Forward: CGCTTTTTGCTTTTGGTGATTC<br>Reverse: AACCTTCGGCAACTATATCGGT
CYP77A6	Forward: CGCTGCGTTTTGTATTCTTTTG<br>Reverse: AACTTGTTCACAACGAACCTCG
ACS2	Forward: ACATCGCTAATTTCCAAGACTACCA<br>Reverse: CTCTATCAAATGCGGCATAGTACG

Average rosette leaf numbers of WT and *GsWRKY20*ox plants at the time of flowering under GA3 treatment.

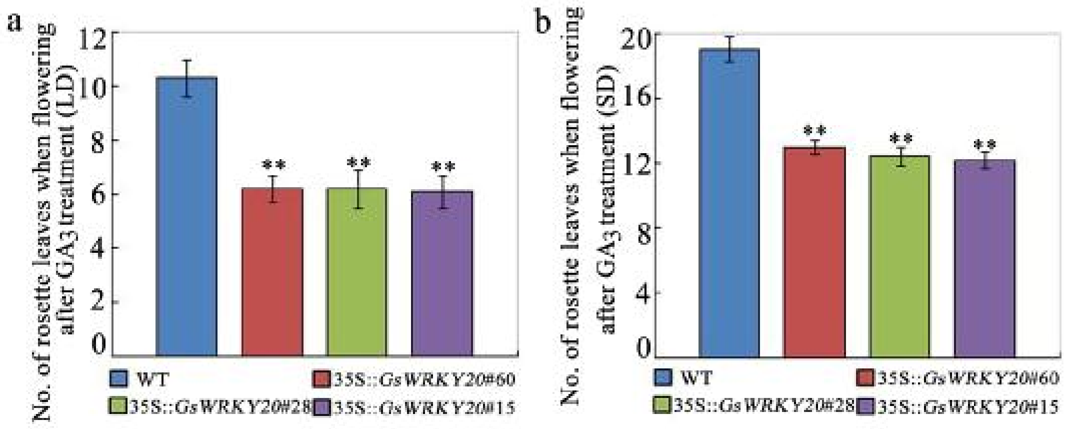

All values are means (±S.E.) from three independent experiments (at least 30 seedlings per experiment). Data were analyzed statistically using the t-test, double asterisks indicate significant differences from the corresponding WT at $P < 0.01$.
